# Change in precipitation pattern over South Asia in response to the trends in regional warming and free-tropospheric aerosol loading

**DOI:** 10.1038/s41598-024-64842-7

**Published:** 2024-06-24

**Authors:** Soumyajyoti Jana, Mukunda M. Gogoi, S. Suresh Babu

**Affiliations:** Space Physics Laboratory, Vikram Sarabhai Space Centre, ISRO, Thiruvananthapuram, 695 022 India

**Keywords:** Climate sciences, Atmospheric science

## Abstract

Spatial and temporal shifts in rainfall patterns over South Asia and the adjoining Seas during the pre-monsoon season have been observed over the past 2 decades from 2000 to 2019. Aerosol particles suspended above the boundary layer are a contributing factor to these changes. These particles not only alter cloud characteristics, but also diminish the lapse rate, thereby suppressing convective activity, leading to precipitation anomalies. Over the past 2 decades, high-rainfall regions have experienced declining rainfall, while low-rainfall regions have received increased rainfall. Coinciding with notable anomalies in precipitation, contrasting trends in aerosol optical depth, particularly due to absorbing aerosols in the elevated regions of the atmosphere, are seen. Apart from aerosols, several factors are considered that are critical in modifying precipitation patterns over the study region, such as water vapor content, convective processes, and lower-level relative humidity. We observed a potential transport of excess water vapor by ambient circulation from the oceanic regions having reduced rain, such as Bay of Bengal and the Arabian Sea, to higher latitudes enabling precipitation anomaly at distant locations.

## Introduction

Increasing sea surface temperatures (SST) due to global warming have an impact on the distribution of precipitation globally^1–4^. A warm ocean can lead to an enhancement of extreme precipitation and a decline in light and moderate rainfall, especially in regions where SST warms most^1,5^. Over the Indian Ocean, the warming trend in SST has been quite visible since the 1950s^6^. These changes have been seen as an increase in precipitation in the high-rainfall regions in the tropics^5,7^. The accelerated warming of the Arabian Sea (AS) since the 1995s is creating a significant influence on intense cyclones over the Arabian Peninsula and the Indian subcontinent^6,8,9^. Alongside the warming of the surrounding Seas, the trend and magnitude of warming over India in the last century are quite consistent with the global trend and magnitude^10,11^. According to Kothawale et al.^12^, the average, maximum, and minimum temperatures across India experienced a rise of 0.51, 0.71, and 0.27 °C per century, respectively, from 1901 to 2007. Furthermore, their research indicated a trend of accelerated warming during the latter period from 1971 to 2007. Several other studies predict a global surface temperature increase of 1.4–5.8 °C by the end of the twenty-first century and this is expected to increase the intensity and frequency of extreme weather events like heat waves, droughts, floods, and wildfires^13–16^. Numerous studies have explored the repercussions of climate change on extreme weather occurrences^15,17–20^.

In view of the above, present study examines the changes in pre-monsoon (March, April, and May) rainfall from 2000 to 2019 to understand the impact of climate change. The pre-monsoon period in India is a crucial prelude to the southwest summer-monsoon season, influencing agricultural practices and water resource management^21^. In the agriculture-prone areas of India, pre-monsoon rainfall plays a vital role in the Eastern, Coastal, and Peninsular regions^22^. On average, pre-monsoon receives about 10–11% (110–120 mm) of total rainfall over India as reported by the Indian Meteorological Department. More to it, the intra-seasonal variation in rainfall is quite significant. The variation in pre-monsoon rainfall may impact the near-surface temperature, which can have an influence on the vertical mixing and convective process, creating anomalies in the precipitation-temperature feedback mechanism. However, the study on pre-monsoon rainfall is inadequate with most of them showing trends in specific areas and periods. For example, Sadhukhan et al.^23^ studied the pre-monsoon rainfall over Gangetic West Bengal and its neighbourhood from 1901 to 1992 reporting no long-term trend other than indicating short-term fluctuations. Guhathakurta & Rajeevan^24^ reported a trend of decreasing pre-monsoon rainfall in most subdivisions in the country between 1901 and 2003. A trend analysis by Kumar et al.^25^ from 1871 to 2005 for 135 years reported that there is an increasing trend in pre-monsoon rainfall on a national scale.

In the pre-monsoon months, the Indian subcontinent shows the highest temperature and a low-pressure region which controls the movement of ITCZ towards the north. Kothawale et al.^26^ analysed the trends in daily temperature extremes in the pre-monsoon season over India and reported that the frequency of hot days and nights has increased while cold days showed decreasing trends. Thus, there is a need to identify the recent changes in pre-monsoonal rainfall over India, which are extremely crucial in the wake of climate change. The change in the pre-monsoon rainfall distribution over the Indian subcontinent concerning the effects of atmospheric aerosols is also lacking. It is reported that the decreasing pre-monsoon rainfall over central India is due to the reduction in convective activity^24^ which may be caused by light absorbing elevated aerosols over this region.

In this study, the change in pre-monsoon precipitation over South Asia is explained, elucidating the role of atmospheric dynamical processes and the impact of atmospheric aerosols.

## Results and discussions

### Changes in pre-monsoon rainfall over South Asia

**Figure 1:**
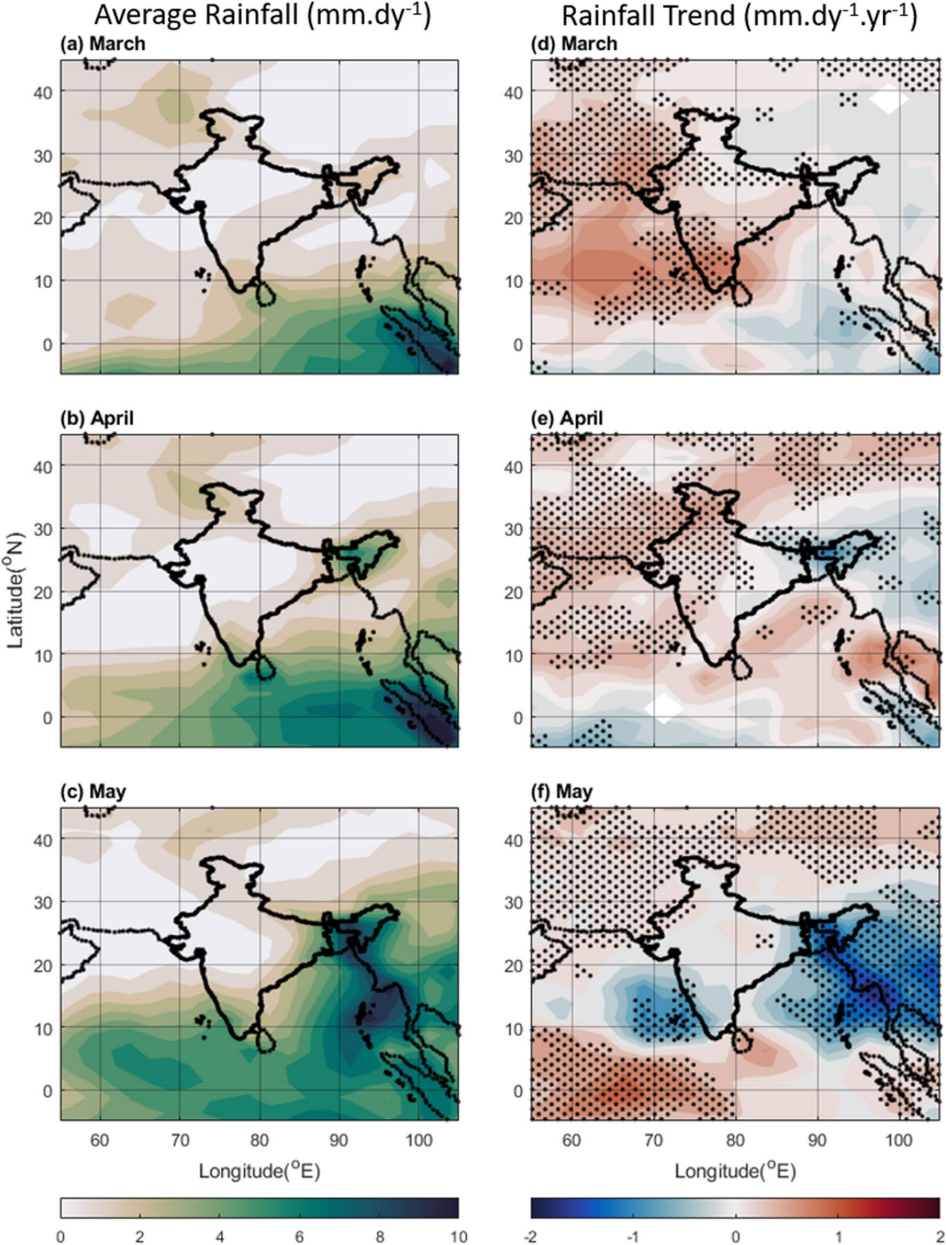
Average rainfall (mm.dy^−1^) in pre-monsoon months of (**a**) March, (**b**) April and (**c**) May during 1981–2010, and trend in rainfall anomaly (calculated from 2000 to 2019) during (**d**) March, (**e**) April, and (**f**) May. The dots correspond to > 90% significance level using the Mann–Kendall test. The Figure is made using MATLAB version 9.13.0 (R2022b), The MathWorks Inc. https://www.mathworks.com.

**Figure 2:**
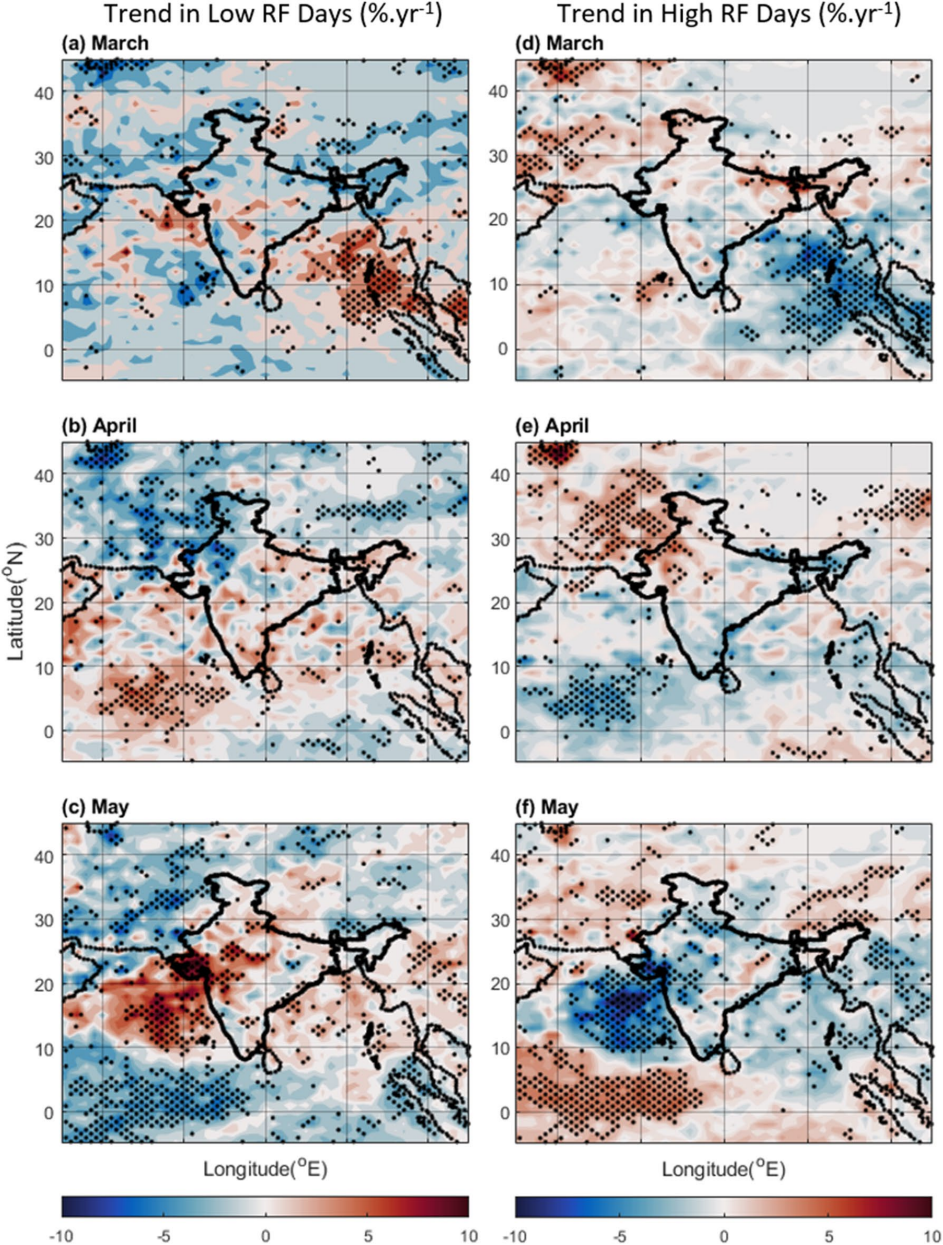
Change in the number of days (in percentage) with rainfall < 2 mm.day^−1^ (low rainfall days) in (**a**) March, (**b**) April, and (**c**) May and change in the number of days (in percentage) with rainfall > 6 mm.day^−1^ (high rainfall days) in (**d**) March, (**e**) April, and (**f**) May. The dots correspond to > 90% significance level using the Mann–Kendall method. The Figure is made using MATLAB version 9.13.0 (R2022b), The MathWorks Inc. https://www.mathworks.com.

This study analyses average pre-monsoon rainfall during 1981 to 2010 and anomaly trends from 2000 to 2019 (Fig. 1) over South Asia and the adjoining Seas, focusing on changes in both total precipitation and frequency of high and low rainfall days (Fig. 2). Our findings reveal a remarkable redistribution of rainfall during the pre-monsoon months. Climatologically, regions like the eastern Bay of Bengal (BoB), the Myanmar Coast (MC), Northeast India (NEI), and Bangladesh receive the bulk of pre-monsoon precipitation (~ 6–10 mm.dy^−1^) (Fig. 1 left panel), experiencing peak rainfall in May. However, our analysis demonstrates a notable decline in rainfall over these traditionally wet regions, particularly the NEI, which witnessed a decrease of ~ 1.0 mm.dy^−1^.yr^−1^ (Fig. 1 right panel). This decline is primarily associated with a reduction in the frequency of high rainfall days in April (Fig. 2 right panel), suggesting a shift from intense downpours to less frequent, lighter events. Conversely, North-western regions (NWR) near North West India, typically arid during this period, have experienced a surprising increase in pre-monsoon rainfall (Fig. 1). Notably, NWR received an additional rainfall of 0.25 to 0.5 mm.dy^−1^.yr^−1^ (Fig. 1), accompanied by a slight increase in high rainfall days in April (Fig. 2). At this juncture, it is to be noted that the “surprise increase in pre-monsoon rainfall” for NWR is used as the long-term average rainfall over such region is, in general, low (~ 2 mm.dy^−1^), but found to experience a rise in rainfall during 2000–2019. Conversely it has been observed that some of the high rainfall areas (such as NEI) are now getting less rainfall. Earlier studies have reported that with the increase in water vapor in the atmosphere, the wet regions will be wetter, and dry regions will be drier^19^. However, the present study shows that this is not the case always for some of the study regions.

The BoB and AS also exhibit interesting trends. Both regions are experiencing a notable decrease in rainfall during May, exceeding 1.5 mm.dy^−1^.yr^−1^ (Fig. 1 right panel). This decline is linked to a reduction in high rainfall days (~ 3% per year) (Fig. 2 right panel). The reduction in high rainfall days is higher over AS (Fig. 2), especially near the Lakshadweep region (2–10% per year during April and May) with the highest reduction in May (Fig. 2e,f). Interestingly, the AS and BoB exhibit increasing trends in total rainfall in March and April, but this is solely due to an increase in low rainfall days (Fig. 2a,b), highlighting a change in the type of precipitation.

Further analysis reveals an inverse relationship between the frequency of high and low rainfall days across the region (Fig. 2). As the number of high rainfall days decreases in certain areas, the number of low rainfall days often increases, and vice versa. This change in the precipitation scenario has been investigated in the present study given all possible influencing parameters, such as the properties of clouds and the associated outgoing longwave radiation (OLR). Lower OLR values particularly indicate the presence of tall, rain-laden clouds^27–29^. By analysing OLR patterns, we can gain valuable insights into the underlying factors driving the observed shifts in pre-monsoon rainfall. In brief, the results highlight significant spatio-temporal changes in pre-monsoon rainfall patterns in recent decades.

### Change in cloud formation

**Figure 3:**
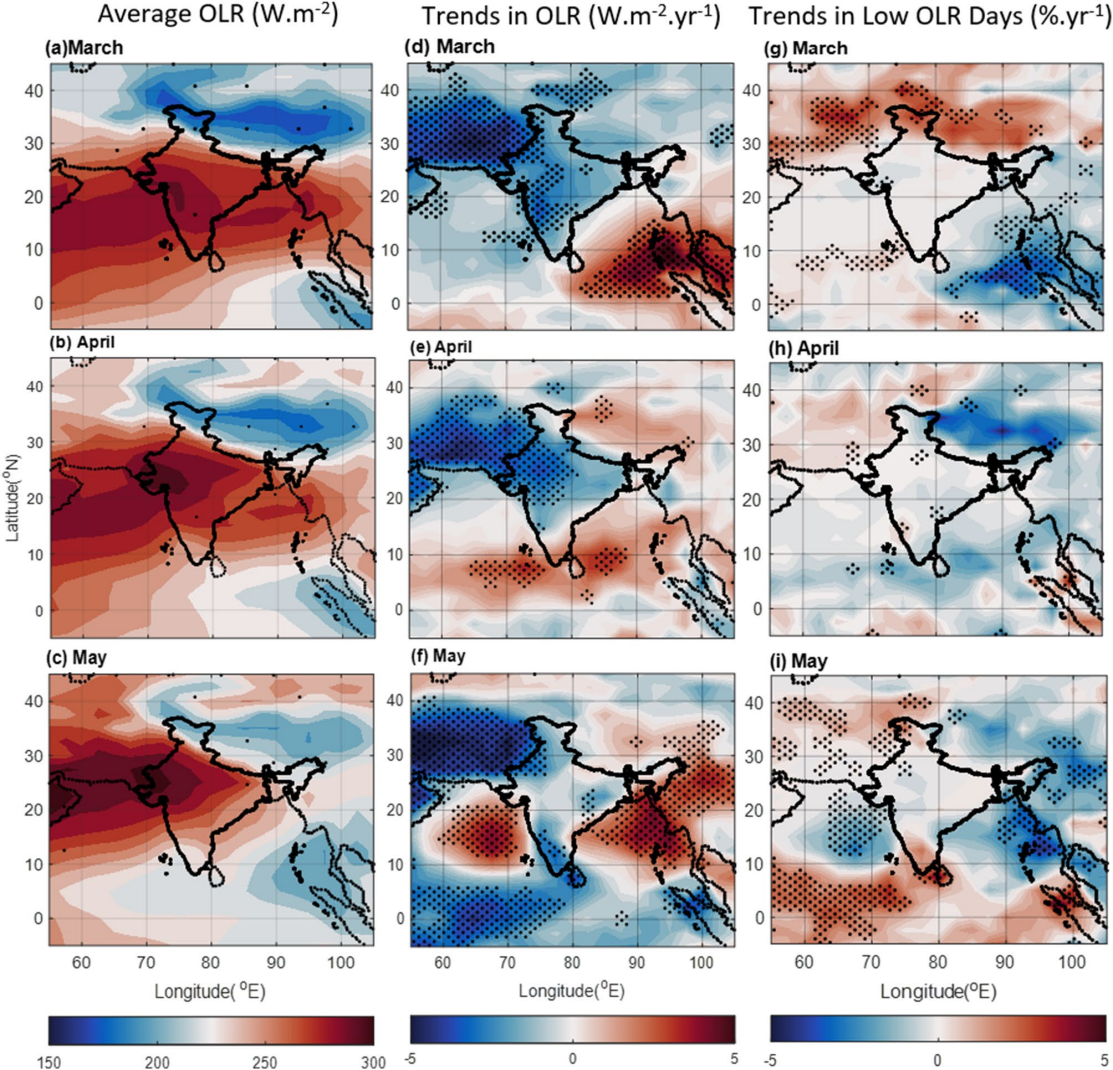
Average OLR (W.m^−2^) in pre-monsoon months of (**a**) March, (**b**) April, and (**c**) May during 1981–2010 and trend in OLR anomaly (calculated from 2000 to 2019) during (**d**) March, (**e**) April, and (**f**) May. The dots correspond to > 90% significance level using the Mann–Kendall method. Change in the number of days (percentage) with low OLR (< 200Wm^−2^) in (**g**) March, (**h**) April, and (**i**) May from 2000 to 2019. The Figure is made using MATLAB version 9.13.0 (R2022b), The MathWorks Inc. https://www.mathworks.com.

Figure 3 presents the regional picture of OLR during the pre-monsoon months. A long-term average of OLR (1981–2010) has been presented in Fig. 3 (left panel). The trend analysis of OLR anomaly from 2000 to 2019 is shown in the middle panel, which clearly reveals the association between cloud formation and rainfall variations. It is observed that the regions consistently exhibiting low OLR (< 200 Wm^−2^; Fig. 3 left panel), indicative of deep convective cloud systems^28^, receives high pre-monsoon rainfall (Figs. 1 left panel). However, the OLR trend unveils a different story. The NEI, BoB, and MC, traditionally wet regions, show a significant decline in convective cloud occurrence, evidenced by increasing trend in OLR. This decline corresponds to the observed decrease in rainfall (Fig. 1 right panel). On the other hand, NWR presents a contrasting picture. Here, we observed an increase in cloud formation evidenced by the decreasing trend in OLR.

To ensure this trend wasn't driven by infrequent, exceptionally low OLR events, we utilized daily OLR data. The focus shifted to the number of "low OLR days" (< 200 W/m^2^), representing days with large convective clouds (Fig. 3 right panel). NWR exhibited a consistent increase in low OLR days across all pre-monsoon months, peaking in March (~ 2% increase per year). This closely aligns with the observed rise in rainfall in this region (Fig. 1 right panel). Conversely, the NEI showed the mixture of both increasing and decreasing trends in the low OLR days, while BoB and AS witnessed a significant decrease in low OLR days, indicating reduced convective cloud activity in May (Figs. 3i). This explains the declining rainfall (Fig. 1 right panel) over these regions. Regions experiencing long-term declines in rainfall (Fig. 1 right panel) generally show a corresponding decrease in low OLR days (Fig. 3 right panel), further elucidating the link between cloud patterns and rainfall variations. To complete the picture, cloud fraction data from MODIS was analyzed (Fig. S2). This helps differentiate generalized cloud cover from highly convective, rain-producing clouds. Figure S2 right panel shows decreasing trend in cloud cover over traditionally high-rainfall regions like the BoB, and AS. Interestingly, an increasing trend in cloud fraction over NEI in May coincides with the increasing trend in high rainfall days over the region. In contrast, NWR exhibits an increase in cloud fraction. This, coupled with increasing trend in low OLR days and precipitation over NWR (Figs. 3 right panel and 1 right panel), suggests a potential precipitation efficiency in this region. Overall, Fig. 3 presents compelling evidence of changing pre-monsoon cloud patterns across India, directly impacting regional rainfall distribution. Understanding these intricate patterns is crucial for water resource management, disaster preparedness, and agricultural planning in a changing climate.

### Aerosol scenario

Figures 1 and 2, combined with OLR and cloud fraction data (Figs. 3, S2), indicate changes in cloud formation, growth, and precipitation patterns over the BoB and AS, as well as over NEI and NWR during the past 2 decades. While moisture content, cloud condensation nuclei (CCN), and atmospheric buoyancy are the primary drivers of cloud formation, the role of aerosols influencing the convective processes cannot be ignored^30–32^. While, sea salt aerosols, for instance, act as efficient CCNs and facilitate precipitation^33,34^, light-absorbing aerosols like dust, polluted dust and smoke at elevated heights can impede cloud growth by reducing temperature lapse rates and hindering convection^35,36^.

Elevated aerosol layers have been reported in many studies during the pre-monsoon season (March–May) over the Indian region^36–40^. The elevated layers are formed in two main ways. Firstly, strong convection can push pollutants from the surface layer up to the free troposphere. Secondly, wind-driven advection of aerosol from one place to another above the boundary layer. Over the Indian region, the elevated layers of aerosols are usually formed during the pre-monsoon season due to strong convective activity^37,41^. If these lifted aerosols are absorbing in nature, they trap radiations, reducing the natural decrease in temperature lapse rate^35,36^. Furthermore, air flowing outwards from landmasses in India can carry these pollutants long distances above the boundary layer, potentially reaching nearby Bays. This phenomenon may weaken atmospheric convection, thereby affecting cloud formation and rainfall over the oceanic regions.

**Figure 4:**
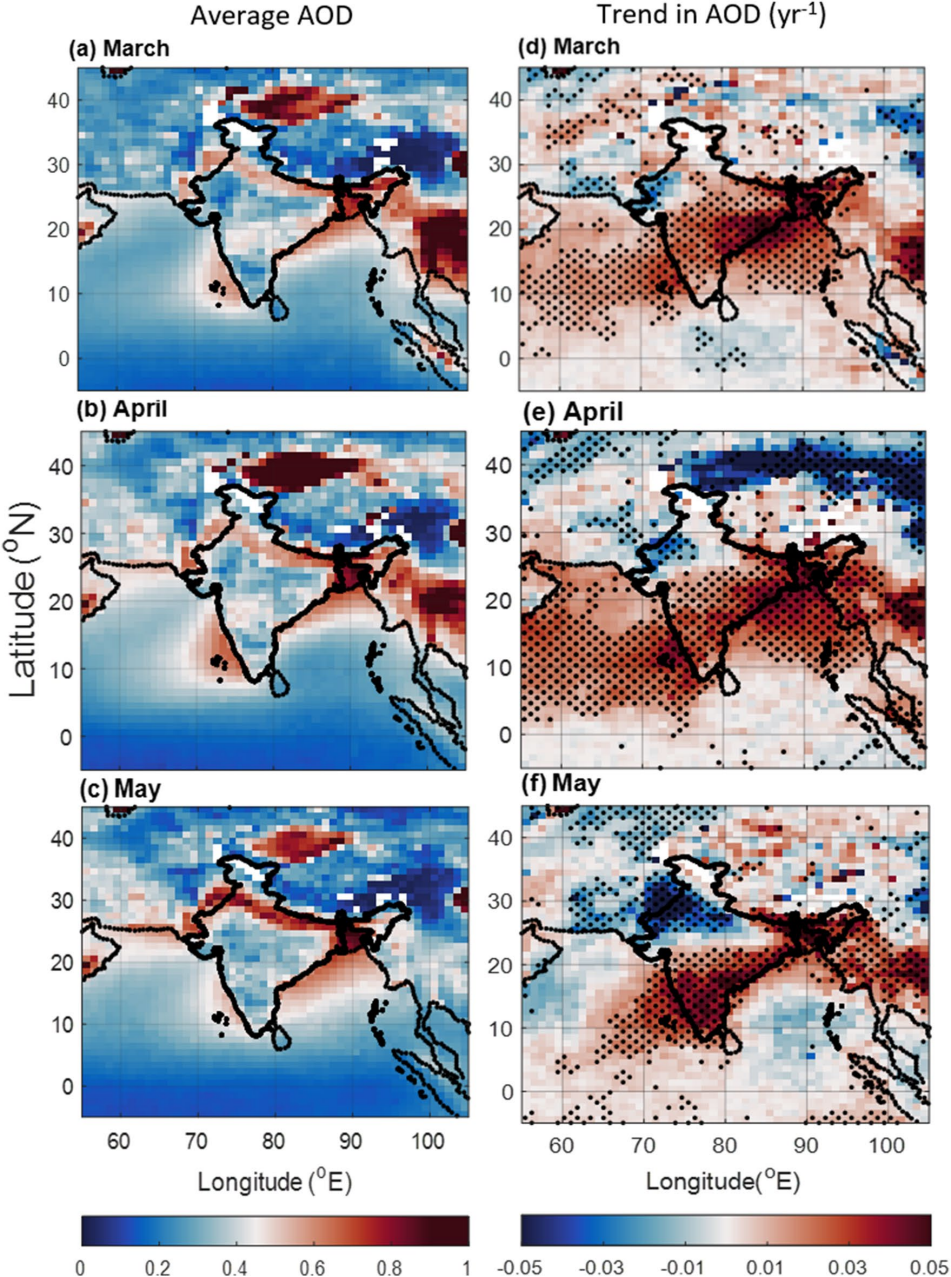
Average AOD in pre-monsoon months of (**a**) March, (**b**) April, and (**c**) May during 2000–2019 and trend in AOD during (**d**) March, (**e**) April, and (**f**) May. The dots correspond to > 90% significance level using the Mann–Kendall method. The Figure is made using MATLAB version 9.13.0 (R2022b), The MathWorks Inc. https://www.mathworks.com.

To elucidate the aerosol influence, we have utilized MODIS Aerosol Optical Depth (AOD) at 550 nm for 2000–2019 (Fig. 4). The AOD reveals a significant increasing trend over NEI, as well as over BoB and AS (~ 0.04 yr^−1^) in March and April, reflecting aerosol outflow from the Indian subcontinent (Fig. 4d,e). Notably, a slight decrease in AOD is observed over the south BoB (− 0.02 yr^−1^) in May, although it remains statistically insignificant (Fig. 4f). However, NWR shows a decreasing trend in AOD with the maximum decrease in the month of May (− 0.045 yr^−1^). Further investigation using CALIPSO data for 2000–2018 showed significant regional variability in the vertical distributions of different aerosol types, such as: Dust, Polluted dust (dust mixed with smoke or other non-depolarizing aerosols in CALISPO measurements^42,43^) and, Smoke (from biomass burning/forest fires).

The light-absorbing properties of aerosols, particularly, polluted dust, and smoke, significantly impact vertical transport and convective available potential energy (CAPE), hindering cloud formation^35^. While dust concentrations (integrated between 1.6 to 4 km) exhibit complex spatiotemporal patterns with a decreasing trend above the boundary layer over most of the study regions in May (Figs. S3 right panel), polluted dust and smoke show increasing trends, especially over coastal regions (Figs. S4, S5 right panels), which can influence the temperature lapse rate. Polluted dust over the Indian mainland is highest in April, but displays a gradual increase over the oceanic regions from March to May (Fig. S4 right panel). Smoke aerosols, with seasonal peaks in March, also exhibit increasing trends, primarily over coastal areas of BoB and AS (Fig. S5). Notably, smoke aerosols show an increasing trend in the NEI in May (Fig. S5f.), similar to that of dust. These findings highlight the probable growing influence of absorbing aerosols on the change in cloud formation in the BoB and AS, as well as in distinct regions of the Indian mainland.

As ocean typically experiences weaker convection than that over the land^44^, the weakening effect of aerosols on convection over ocean (like Bays) may be more significant compared to land, especially in comparison to the landmasses of arid types. Thus, the same elevated aerosol layer can have a more substantial impact on weakening convection over the oceanic regions. Additionally, recent years have seen an increase in absorbing aerosols above the marine boundary layer of the AS and BoB, as well as in the free-tropospheric regions of NEI (Figs. S4, S5). This is indicative of the influence of elevated layers of absorbing aerosols on natural temperature lapse rate, weakening convection more than what is observed over arid land masses. This difference in convection weakening could lead to an opposite rainfall trend as is seen in the present findings. We observed an increasing trend (~ 0.04 yr^−1^) in aerosol levels (AOD) over the BoB, AS and NEI (Fig. 4), which coincides with a decrease in rainfall there. This hints at a potential link between the increase in aerosols and the decrease in rainfall observed over these regions.

These findings also suggest a growing influence of aerosols on cloud formation over BoB and AS, as well as in NEI. Especially, the light-absorbing aerosols could potentially suppress convection and could impact regional precipitation patterns. However, to fully comprehend their impact on the hydrological cycle, the increasing burden of light-absorbing aerosols requires further investigation, particularly into their association with water vapor distribution.

### Water vapor distribution

**Figure 5:**
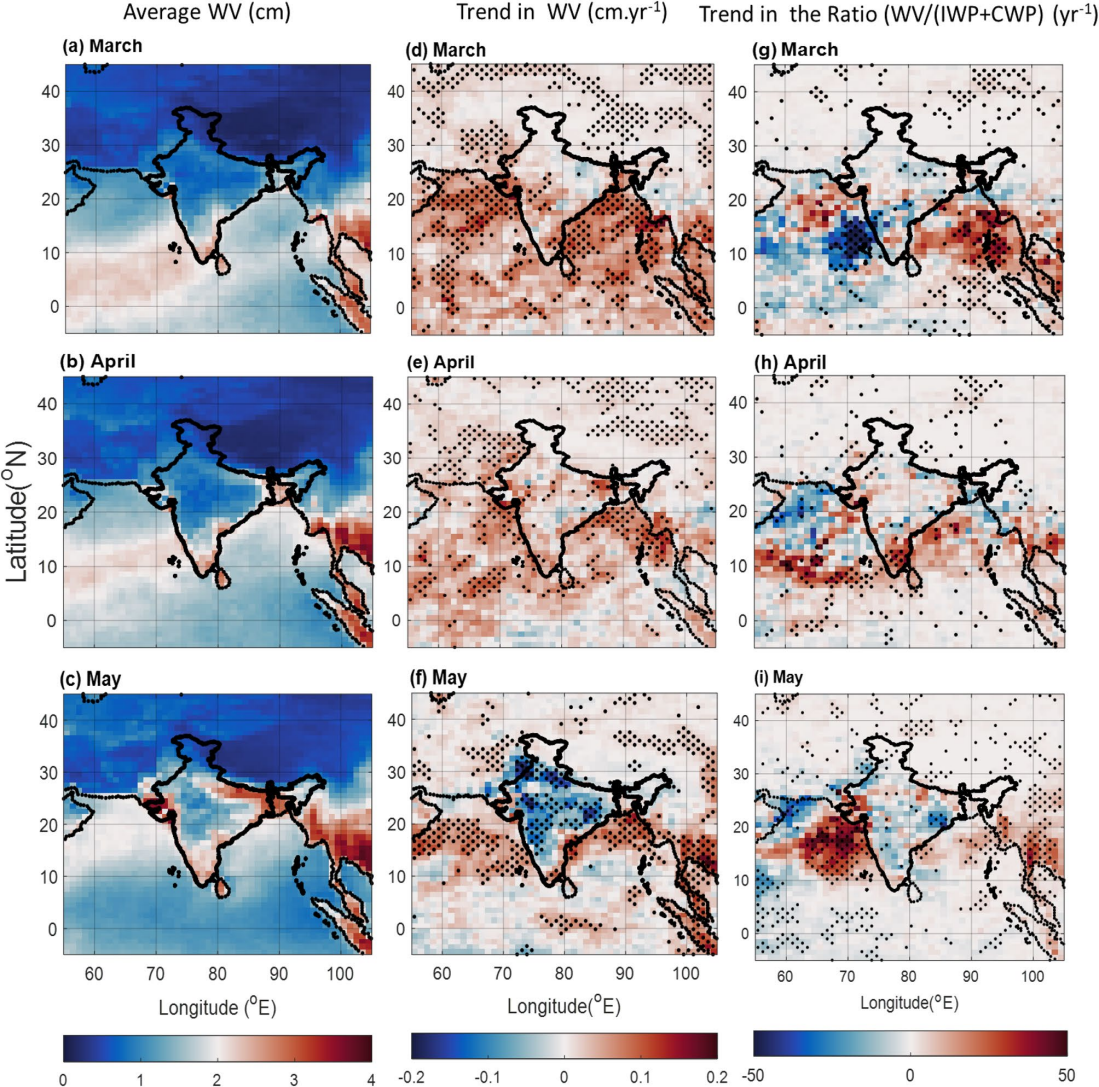
Average near-infrared cloudy column water vapor (cm; mean of daily mean) from MODIS for (**a**) March, (**b**) April, and (**c**) May during 2000–2019, and the trend in water vapor during the same period for (**d**) March, (**e**) April, and (**f**) May. The dots correspond to a > 90% significance level using the Mann–Kendall method. Trend in the water vapor to cloud hydrometeor (IWP + CWP) ratio during 2000–2019 for (**g**) March, (**h**) April, and (**i**) May for MODIS partly cloudy pixels. The dots correspond to a > 90% significance level using the Mann–Kendall method. The Figure is made using MATLAB version 9.13.0 (R2022b), The MathWorks Inc. https://www.mathworks.com.

Water vapor, the fuel for precipitation, has risen over the BoB and AS during 2000–2019 (Fig. 5, middle panel). This increase is particularly evident in May (Fig. 5f), during which most of the Indian regions show a decrease (Fig. 5f). On the other hand, a positive trend in WV is seen over NWR and NEI. This variability in WV could be due changes in water vapor transport (Held & Soden, 2006).

The association between water vapor, cloud and precipitation are further complicated by aerosols. While rising water vapor suggests the potential for increased cloud and rainfall, aerosol concentration has also increased in recent years. The "indirect effect" of aerosols can hinder larger/ stable cloud formation and precipitation, even in the presence of abundant water vapor. To analyse this, we examined cloud liquid water content (CWP) and ice content (IWP), combined as a measure of total cloud hydrometeors (Fig. 5, right panel). We then calculated the ratio between water vapor and cloud hydrometeors, providing an indicator of vapor-to-hydrometeor conversion efficiency. The spatial distribution of cloud hydrometeors shows a complex picture. In March, AS near the western coast showed a significant increase in hydrometeors alongside high aerosol concentrations (Figs. 4d, 5g). This may highlight the less significant effect of aerosols on precipitation, as the overall the precipitation over the region shows an increase (Fig. 1d). Conversely, BoB exhibits a decrease in cloud hydrometeors, coinciding with reduced precipitation and potentially linked to the increased presence of light-absorbing aerosols above the boundary layer reducing convective growth (Figs. S4, S5).

In the month of May, cloud hydrometeors show significant decreasing trend over AS and moderate decrease over the BoB. During this month, the AS shows significant increase in polluted dust, and smoke (Figs. S4, S5). Polluted dust also shows increasing trends over the BoB (Fig. S4). This might have affected cloud formation and rain over these regions as is evident from Figs. 1, 3. Over the land regions, smoke and dust aerosols show increasing trends over NEI, while the trends of absorbing aerosols are not significant over the NWR (Figs. S3, S4, S5). Thus, the rise in rainfall over NWR could be attributed to efficient vapor-to-hydrometeor conversion in the presence of abundant water vapor (Figs. 1 right panel, 5, 4 right panel).

These findings underline the crucial role of aerosols in modulating precipitation, particularly over the Seas and distinct regions of South Asian landmasses. This emphasizes the need to further investigate the combined effects of changing water vapor, aerosols, and oceanographic parameters like sea surface temperature (SST) and ocean heat content (OHC) on regional precipitation patterns. Analyzing SST and OHC within 300 m depth is crucial, as they not only influence cloud formation and precipitation over the sea but also impact land precipitation through water vapor transport^45–47^.

### Ocean parameters

SST plays a crucial role in shaping regional precipitation patterns. Figure S6 (left panel) reveals an increasing trend in SST near coastal regions in all months, which might inevitably boost evaporation from the sea surface. Shallow sea depths near the coast contribute to larger variations in SST in these locations. Previous studies suggest that SST increase up to 30 °C enhances precipitation. Interestingly, recent research^48^ indicates a positive linear relationship between SST and precipitation even above 30 °C. Figure S6 (left panel) shows a positive SST trend across the study region. The AS experiences more severe warming trend than the BoB, especially in March and April (Fig. S6 left panel). However, an interesting anomaly in the north BoB is observed between March and May. Earlier reports suggest that aerosol outflow over this region can reduce incoming solar radiation, thereby cooling the sea surface in that region^49^. Additionally, at higher altitudes, anthropogenic aerosols have a higher heating efficiency than dust^50^ and can cause surface dimming. This is clearly visible in our present study. During March, when aerosol outflow increases (i.e., increasing trend in AOD over BoB) significantly (Fig. 4d), the SST for the north BoB doesn't show an increasing trend (Fig. S6a). However, in May, when this aerosol outflow is not significant (Fig. 4f), SST shows increasing trend in the north BoB (Fig. S6c). Additionally, while May shows highest AOD over BoB (Fig. 4f), CALIPSO's species analysis reveals dust aerosols as the primary contributor. However, the trend in dust AOD shows a decrease over BoB, while AOD due to polluted dust and smoke shows an increase, peaking in March and diminishing towards May (Figs. S3f., S4f., S5f.). These observations indicate the possible direct effect of aerosols on the variability in SST over the adjoining seas of South Asia.

Recent studies emphasize ocean heat content (OHC) as a more critical parameter influencing evaporation and associated cloud formation and rainfall^51^. Over the AS, the OHC trend shows a significant increase compared to the BoB, explaining enhanced evaporation over the AS (Fig. S6 middle panel). The lower AOD over the AS compared to the BoB further contributes to increased heating and higher OHC over the AS (Figs. S4, S5).

Increased evaporation also leads to reduced salinity in both the AS and BoB (Fig. S6 right panel). Lower salinity increases the presence of sea salt in the atmosphere, acting as hygroscopic CCNs that efficiently promote cloud formation. This effect is less pronounced over the BoB, likely due to the significant rise in absorbing aerosols, which decrease convective strength as observed in OLR over the past 20 years (Fig. 3 middle panel).

### Meteorological changes

**Figure 6:**
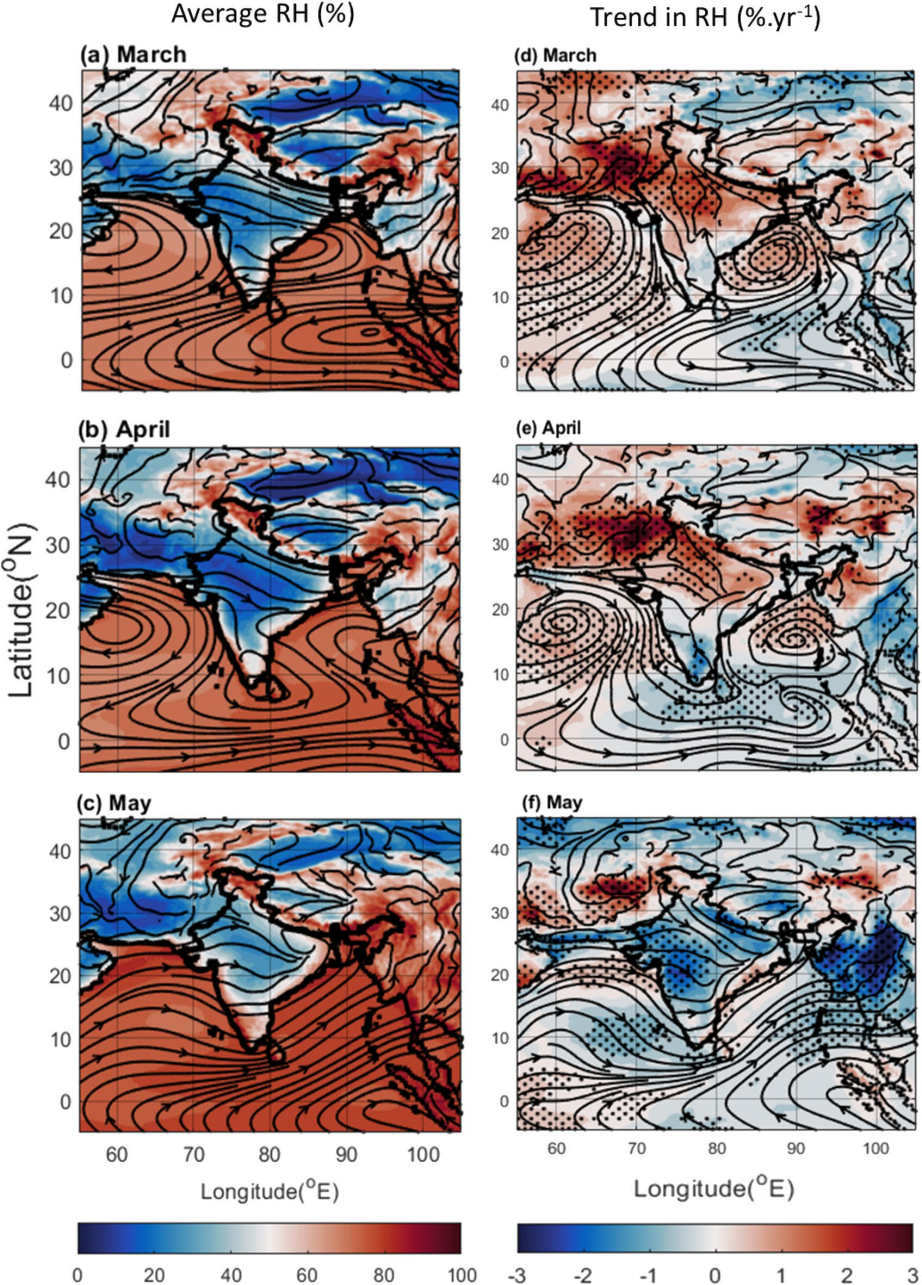
Average RH (%) from ERA-5 for (**a**) March, (**b**) April, and (**c**) May during 2000–2019, and the trend in RH (% yr^−1^) during the same period for (**d**) March, (**e**) April, and (**f**) May. The dots correspond to a > 90% significance level using the Mann–Kendall method. In the case of trends, the streamlines are shown for the year 2019; while the streamlines in the mean plots (left panel) are the average zonal and meridional wind during 2000–2019. The Figure is made using MATLAB version 9.13.0 (R2022b), The MathWorks Inc. https://www.mathworks.com.

**Figure 7:**
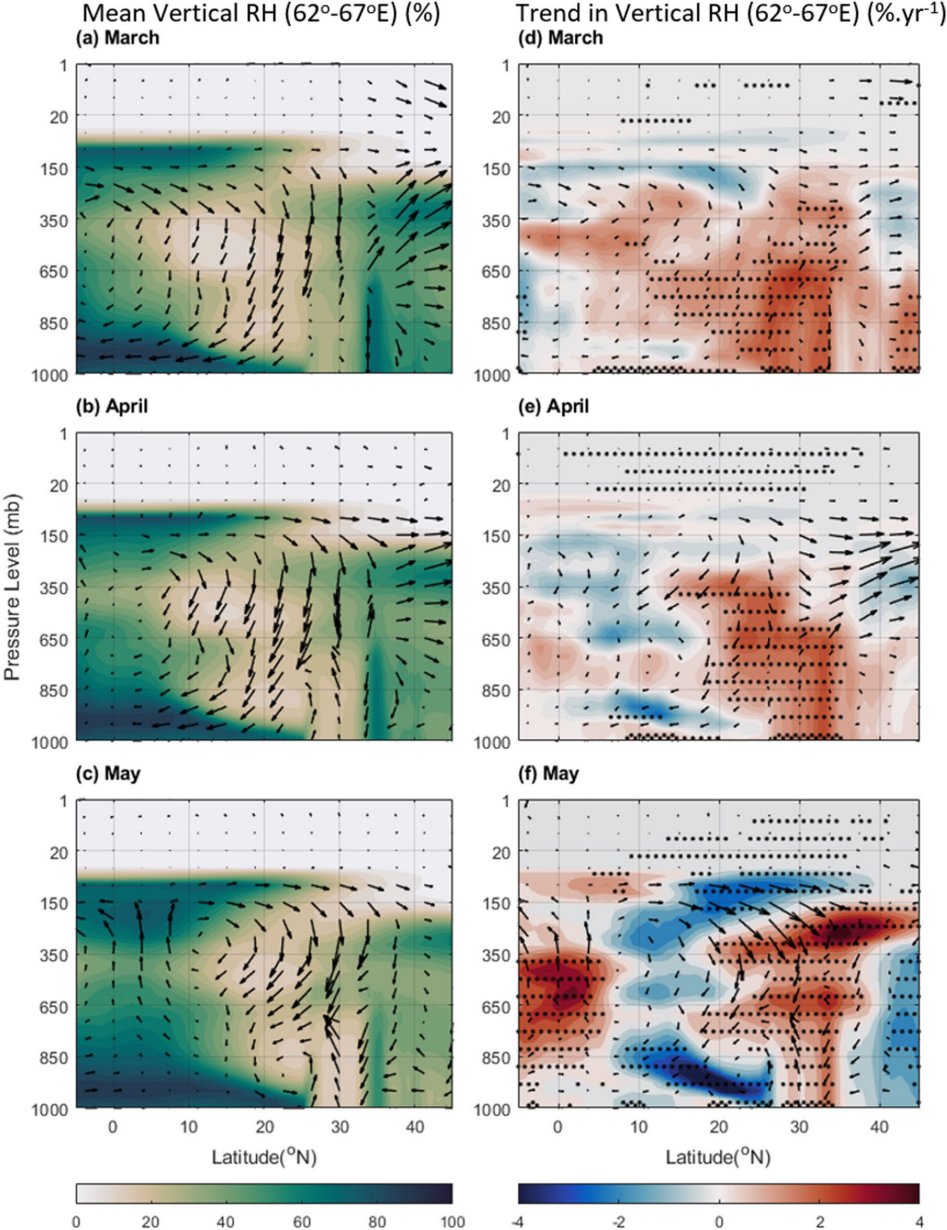
Zonal Average of RH (%) over longitude range of 62-67^°^E (covering AS and NWI) from ERA-5 for (**a**) March, (**b**) April, and (**c**) May during 2000–2019, and the trend in RH (% yr^−1^) during the same period for (**d**) March, (**e**) April, and (**f**) May. The dots correspond to a > 90% significance level using the Mann–Kendall method. In the case of trends, the wind vectors are shown for the year 2019 while the wind vectors in the mean plots (left panel) are the average vertical and meridional wind during 2000–2019. The Figure is made using MATLAB version 9.13.0 (R2022b), The MathWorks Inc. https://www.mathworks.com.

The association between changing ocean conditions (SST and OHC) and meteorological parameters like temperature, wind circulation, and lower-level relative humidity (RH) can impact cloud formation and precipitation patterns over the BoB and AS. Figure 6 depicts the probable relationship between low-level RH and wind circulation at 850 hPa. High RH seen over the oceanic regions of BoB and AS in March and April coincides with high-pressure systems (anti-cyclonic circulation) (Fig. 6a,b), hindering cloud formation as reflected in increased OLR and decreased cloud fraction (Figs. 3, S2). However, the high RH in the month of May coincides with cyclonic circulation over BoB (Fig. 6c). The RH trends with wind streamlines from 2019 further clarify the association. An increasing trend in lower level RH (driven by enhanced evaporation), but coinciding with anti-cyclonic circulations over BoB and AS is indicative of the inhibition of vertical wind movement, restricting cloud formation (Figs. 6d,e, S2b,c). April also shows persistent high pressure over northern BoB (Fig. 6e), suppressing cloud formation (Fig. S2b). The ridge circulation (anti-cyclonic) over NEI in May, along with statistically no significant increasing trend in RH, explains the reduced rainfall in these regions (Figs. 6f, 1f). Conversely, NWR exhibits a significant RH increase along with hints of cyclonic circulation (Fig. 6 right panel). The trough structure (cyclonic) and consistently increasing trend in RH throughout the pre-monsoon months over NWR across months (Fig. 6 left panel) create favourable conditions for cloud formation and rain (Fig. S2 right panel). This suggests the potential transport of excess water vapor from areas with reduced rain, like AS, to regions like NWR. Figure 7 explores this hypothesis by examining the vertical RH profile and wind streamlines over the regions of the AS and NWR (62–67°E, − 5–45°N). As expected, there is high RH distributed latitudinally across the AS and a sharp reduction beyond. Interestingly, the long-term variability in RH shows increasing trends over the AS, as well as over the NWR. This suggests that usual circulation carries more moisture in recent years from the equator northward. Thus, the reduced cloud formation and rain over AS likely contribute to increased RH (Fig. 7 right panel) and potentially increased precipitation in NWR (where aerosol concentrations are also lower, favoring cloud formation).

**Figure 8:**
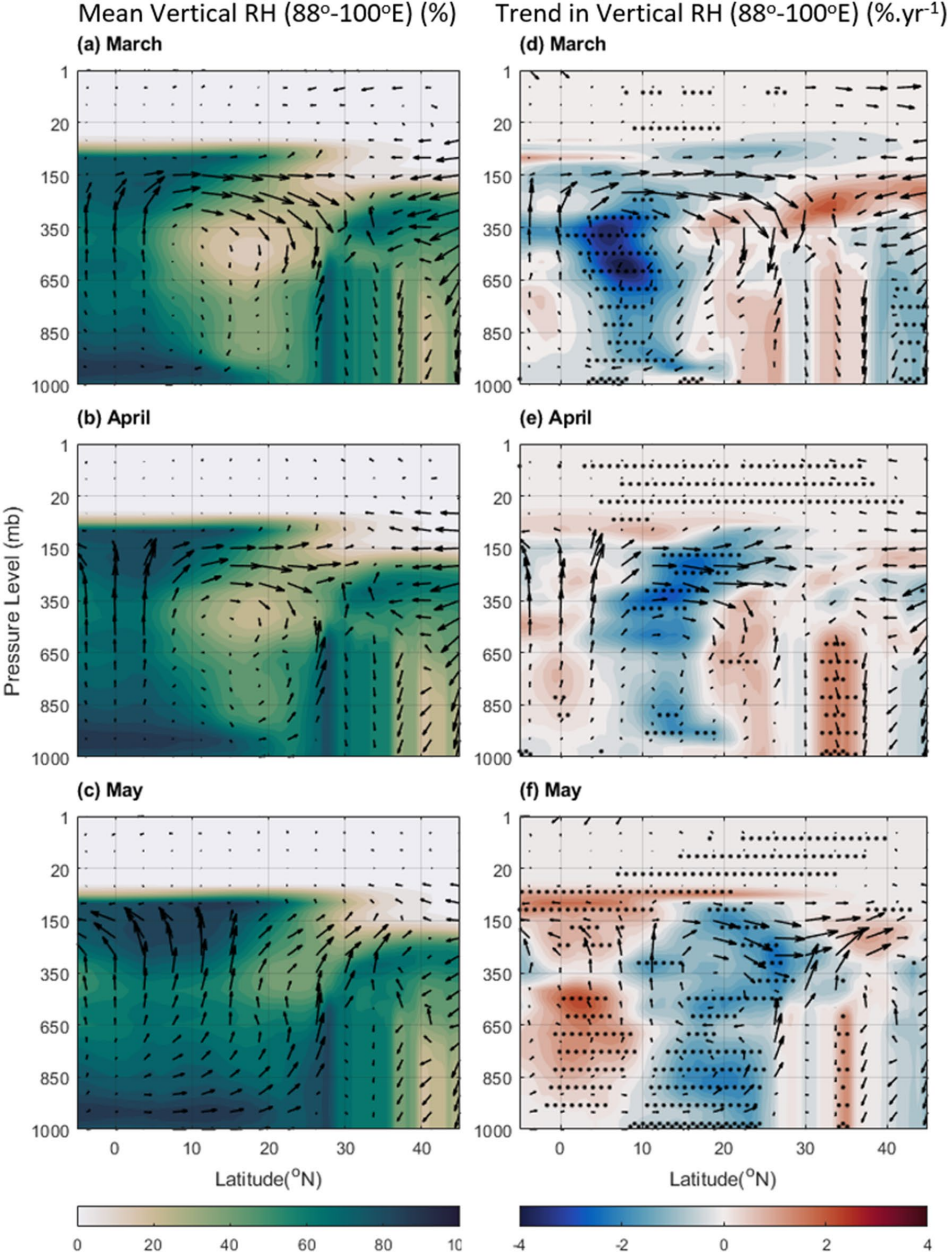
Zonal Average of RH (%) over longitude range of 88-100^°^E (covering BoB, MC, and NEI) from ERA-5 for (**a**) March, (**b**) April, and (**c**) May during 2000–2019, and the trend in RH (% yr^−1^) during the same period for (**d**) March, (**e**) April, and (**f**) May. The dots correspond to a > 90% significance level using the Mann–Kendall method. In the case of trends, the wind vectors are shown for the year 2019 while the wind vectors in the mean plots (left panel) are the average vertical and meridional wind during 2000–2019. The Figure is made using MATLAB version 9.13.0 (R2022b), The MathWorks Inc. https://www.mathworks.com.

Similar analysis over BoB and NEI (Fig. 8) reveals a different scenario. Over this longitude band, the zonal average RH is not only higher over the oceanic regions of BoB, but also shows higher values over the NEI. However, vertical distribution of RH does not show prominent increasing trend over the NEI regions (Fig. 8 right panel). These observations indicate a complex interplay between changing ocean conditions, meteorological parameters, and regional aerosol concentrations shaping precipitation patterns over BoB and AS, as well as that over NWR and NEI. Understanding this interplay is crucial for predicting future changes in regional climate and developing effective water management strategies.

### Statistical evidence

Based on the present analysis, four distinct regions showing significant precipitation anomaly during 200–2019 are considered for Multiple Linear Regression (MLR) analysis. The regions are depicted in Fig. S7, which include (a) Region 1 (90-100E; 6-22N) consisting of BoB, (b) Region 2 (63-77E; 8-20N) consisting of AS, (c) Region 3 (64-74E; 25-34N) consisting of NWR, and (d) Region 4 (88-98E, 23-31N) consisting NEI. The wind (W), relative humidity (RH), temperature (T), and aerosol optical depth (AOD) are taken as independent variables, and precipitation (P) is taken as a dependent variable. MLR provides coefficients for each predictor (wind speed, humidity, temperature, and AOD) and an intercept (i.e., residual) representing the expected precipitation when all predictors are zero. The magnitude of the coefficients indicates the degree of influence on the predicted variable and the sign indicates the nature of the correlation (i.e. positive or negative).

The basic equation for the linear model is given below.

$$P= {\beta }_{0 }+{\beta }_{1 }.W+{\beta }_{2 }. RH+ {\beta }_{3 }.T+ {\beta }_{4 }.AOD+\varepsilon$$

In this equation, the magnitude of ${\beta }_{n}$ is indicative of the impact of the controlling parameters whereas the sign indicates the nature of correlation between dependent (precipitation) and the independent parameters; $\varepsilon$ is the residual. It may be noted that the analysis has been performed with and without AOD. If the inclusion of AOD improves the linear model (in terms of RMSE and *p*-value) then AOD is considered as a significant parameter to influence precipitation. Wind speed (W) is calculated as the magnitude of all three component wind vectors combining zonal (u), meridional (v), and vertical (w); resulting $\text{W}=\sqrt{{(\text{u}}^{2}+}{\text{v}}^{2}+{\text{w}}^{2})$. Based on this, MLR is used to identify the most significant factors influencing precipitation and assess the model's effectiveness. RMSE is calculated from residual’s standard deviation. The statistical significance of the model is chosen based on the low RMSE and *p*-value combination. The *p*-values are considered significant when less than 0.05 (95% significance level).

The coefficients from the linear regression have been given in Table 1 to identify the significant parameters. The significant models are marked in red colour in Table 1. The analysis shows the effect of parameter changes over different regions and months. For Reg. 1 (BoB), the linear model is significant (based on RMSE and *p*-value) for March and May. The precipitation variation is positively influenced by W, RH, and T (i.e. an increase in these parameters causes an increase in precipitation and vice versa) and negatively influenced by AOD in May (i.e. increase in AOD along W, RH and T causes a decrease in precipitation and vice versa). However, the inclusion of AOD improves the linear model in May; indicating the significant role of aerosols in decreasing precipitation over BoB (Fig. 1f). For Reg. 2 (AS) the linear model is significant for March and low-level RH is a major contributor (high value of the coefficient) having positive influence. However, in the case of AS, the inclusion of AOD does not improve the model (RMSE and *p*-value); so, indicating that the precipitation changes are not significantly affected by aerosols. The linear model describes the precipitation well in the case of the Reg. 3 (NWR) in May where the most influencing parameters are W and RH showing positive correlations with precipitation. This suggests again that the increase in rain over this region is related to higher wind flow and probable transportation of more water vapor. Inclusion of AOD does not improve the model over this region; so, the changes in the precipitation are mostly controlled by the background meteorology. Over Reg. 4 consisting of NEI, wind has a significant positive correlation with the precipitation in April. Additionally, the model improves in May after the inclusion of AOD; indicating the role of aerosols in the reduction of rainfall over this region (Fig. 1f). It may be noted here the MLR analysis is made with only four parameters as predictors to keep the process more general, so that the study can hint towards more probable influencing factors. This can be a motivation for more rigorous analysis considering more influencing factors which may result in more reliable quantification.

**Table 1:** Normalised coefficients of the independent variables for multi-linear regression; **a** with AOD and **b** without AOD.

Region	Month	Normalized coefficients				RMSE	*p*
		Wind	RH	T	AOD		
*(a) With AOD*							
Region-1 BoB	March	− 0.05659	0.42253	− 0.47155	0.00440	0.75	0.012
	April	− 0.29731	1.11317	0.49234	− 0.26512	0.89	0.126
	May	0.73464	0.31181	0.25038	− 0.37301	**0.69**	**0.003**
Region-2 AS	March	− 0.15254	0.33508	− 0.27961	− 0.06111	0.85	0.070
	April	0.48989	0.63613	0.08102	− 0.09724	0.96	0.280
	May	− 0.37741	0.10857	− 0.39176	0.15636	0.91	0.150
Region-3 NWR	March	− 0.24265	0.18059	0.16696	0.13013	1.05	0.709
	April	0.20933	0.51961	− 0.00180	− 0.04272	0.97	0.340
	May	0.39487	0.51389	0.06579	− 0.17388	0.67	0.002
Region-4 NEI	March	0.23773	0.92273	0.37767	− 0.08144	0.60	0.001
	April	0.53929	0.30199	0.17845	− 0.54916	**0.68**	**0.003**
	May	0.49638	0.03625	− 0.06053	− 0.84270	**0.75**	**0.012**
*(b) Without AOD*							
Region-1 BoB	March	− 0.05634	0.42356	− 0.46876	0	0.73	0.004
	April	− 0.28971	0.83241	0.22713	0	0.90	0.104
	May	0.53272	0.37206	0.16952	0	**0.76**	**0.008**
Region-2 AS	March	− 0.18306	0.35537	− 0.25417	0	0.83	0.031
	April	0.47925	0.69120	0.11494	0	0.93	0.167
	May	− .33754	0.03903	− 0.37248	0	0.89	0.080
Region-3 NWR	March	− 0.18641	0.24936	0.18969	0	1.02	0.550
	April	0.19266	0.49452	0.01200	0	0.94	0.200
	May	0.49373	0.40832	0.04272	0	0.66	0.001
Region-4 NEI	March	0.23785	0.92587	0.36141	0	0.59	0.001
	April	0.47156	0.41593	0.07187	0	**0.85**	**0.050**
	May	0.07299	0.43416	0.13101	0	**1.03**	**0.593**

It may be noted that a higher absolute value corresponds to higher influence and the sign indicates correlation type (positive/negative). Bold marked months of the corresponding region has significant aerosol influence as RMSE and *p*-value improves after inclusion of AOD.

## Summary and conclusion

Global warming leads to a higher holding capacity of water vapor in the atmosphere. However, it does not necessarily translate to an increased number of heavy rainfall events in an ideal scenario. Firstly, heavy rainfall requires a larger accumulation of water vapor in the cloud (i.e. extended cloud life). The increase in aerosol load leads to increased cloud lifetime. However, as the cloud lifetime increases, the entrainment of air may lead to the dissipation of the cloud^52^. Secondly, the presence of light-absorbing aerosol above the boundary layer hinders convection and, therefore cloud formation and rain. These factors cause rain suppression by aerosols which is a concern in recent times. So, in an ideal scenario where aerosols are less or constant, an increase in water vapor results in a higher number of low rainfall events (warm rain) as aerosols (which act as CCN) help in holding more water vapor and, they grow quickly resulting in rain. Yano and Manzato^53^ concluded in their study that the frequency (number of events) and the intensity (severity) of rain do not follow one another when moisture content increases in the atmosphere. They showed that though the frequency shows increment, the intensity does not. However, if the number of rain events does not change or is even reduced, more water vapor results in extreme events as the water vapor availability per event increases. This also reported in earlier reports suggesting that an increase in water vapor can increase rain over the wetter regions, while dry regions become drier^19^. But this is also not true for all regions. In the present study, we have seen that “dry gets drier, wet gets wetter” scenario is not observed over the Indian region during pre-monsoon from 2000 to 2019. Because many other influencing factors like wind circulation, temperature, and aerosol concentration come into play.

**Figure 9:**
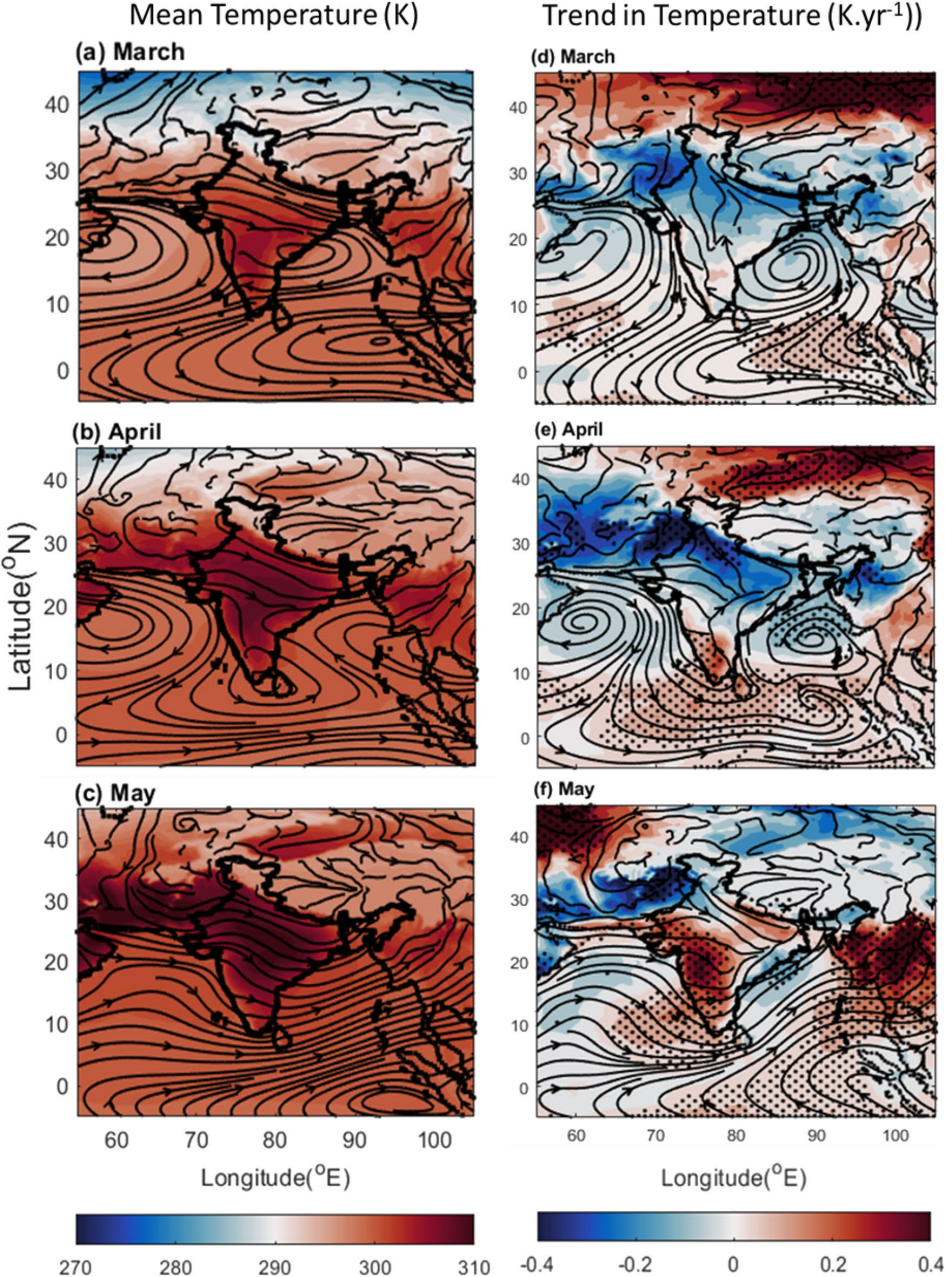
Average of Temperature (K) from ERA-5 for (**a**) March, (**b**) April, and (**c**) May during 2000–2019, and the trend in Temperature (K yr^−1^) during the same period for (**d**) March, (**e**) April, and (**f**) May. The dots correspond to > 90% significance level using the Mann–Kendall method. In the case of trends, the streamlines are shown for the year 2019 while the streamlines in the mean plots (left panel) are the average zonal and meridional wind during 2000–2019. The Figure is made using MATLAB version 9.13.0 (R2022b), The MathWorks Inc. https://www.mathworks.com.

As the Indian subcontinent has witnessed a remarkable transformation in its precipitation patterns over the past 2 decades during the pre-monsoon period, this study investigates the factors driving these changes. The shifts in rainfall distribution are seen primarily over the traditionally wetter regions like the BoB, and NEI experiencing a decline in rainfall, while the Northwest region has seen an increase (Fig. 1 right panel). It can be noted that most of the pre-monsoon rain occurs in May (Fig. 1 left panel) which experiences the most significant change in the precipitation patterns. The precipitation types also changed over time (Fig. 2). The frequency of high-intensity rainfall has decreased, replaced by more frequent low-intensity events. This phenomenon significantly reduced overall rainfall in May over the AS and BoB (Fig. 2). A complex association of factors reveal the following:

1. The ocean parameters like SST and OHC show a significant rise over the present study region which reduces salinity due to the increased evaporation (Fig. S6). Though amplifying water vapor availability, it doesn't directly contribute to enhanced rain in typical pre-monsoon zones due to other influencing factors.
2. The increased presence of aerosol and changing atmospheric circulation in terms of water vapor transportation altered the scenario of precipitation. Over the high rainfall regions mentioned above (mainly MC, BoB, and NEI) lower level RH is reduced (Fig. 6), and above boundary layer anthropogenic aerosol presence increased (Fig. S4, S5) reducing the environmental lapse rate and hence precipitation. Cloud formation and associated convective activity as indicated by the OLR and CF data show that reduction in the rain is associated with reduction in convective clouds. On the other hand, the reduction in aerosol and increase in lower level RH (Figs. 4, 6, 7) gives a favourable condition for the formation of rain over NWR.
3. The change in the meteorology and aerosol environment has an efficient impact on cloud formation which results in precipitation anomaly. This leads to an anomaly in temperature (Fig. 9). The temperature has been found to increase over MC and WC regions which have also experienced a reduction in rainfall in the last 20 years. Whereas, it can be seen that the temperature has decreased over the NWR significantly every month with the highest change in May (− 0.4 K.yr^−1^) (Fig. 9f). This could be associated with the increasing trend in precipitation over NWR (Fig. 1). This kind of temperature reduction weakens the movement of monsoon trough^54^ towards the north as decreasing temperature reduces the strength of the low pressure residing over the land during pre-monsoon (requires a separate study). So, Changes in rainfall distribution are likely to influence temperature patterns, creating a feedback loop affecting overall circulation and precipitation dynamics.

Overall, this study utilized a comprehensive dataset encompassing satellite observations, ground station measurements, and reanalysis products. These datasets covered key parameters like precipitation, temperature, humidity, wind speed, and aerosol optical depth (AOD). The statistical analysis using MLR revealed wind, humidity, temperature, and aerosols significantly influence precipitation patterns. The dominant controlling factors vary by region and month. Aerosols play a pivotal role in suppressing rainfall over the BoB and NEI. The MLR analysis considered potential interactions between variables and assessed the statistical significance of the results.

**Figure10:**
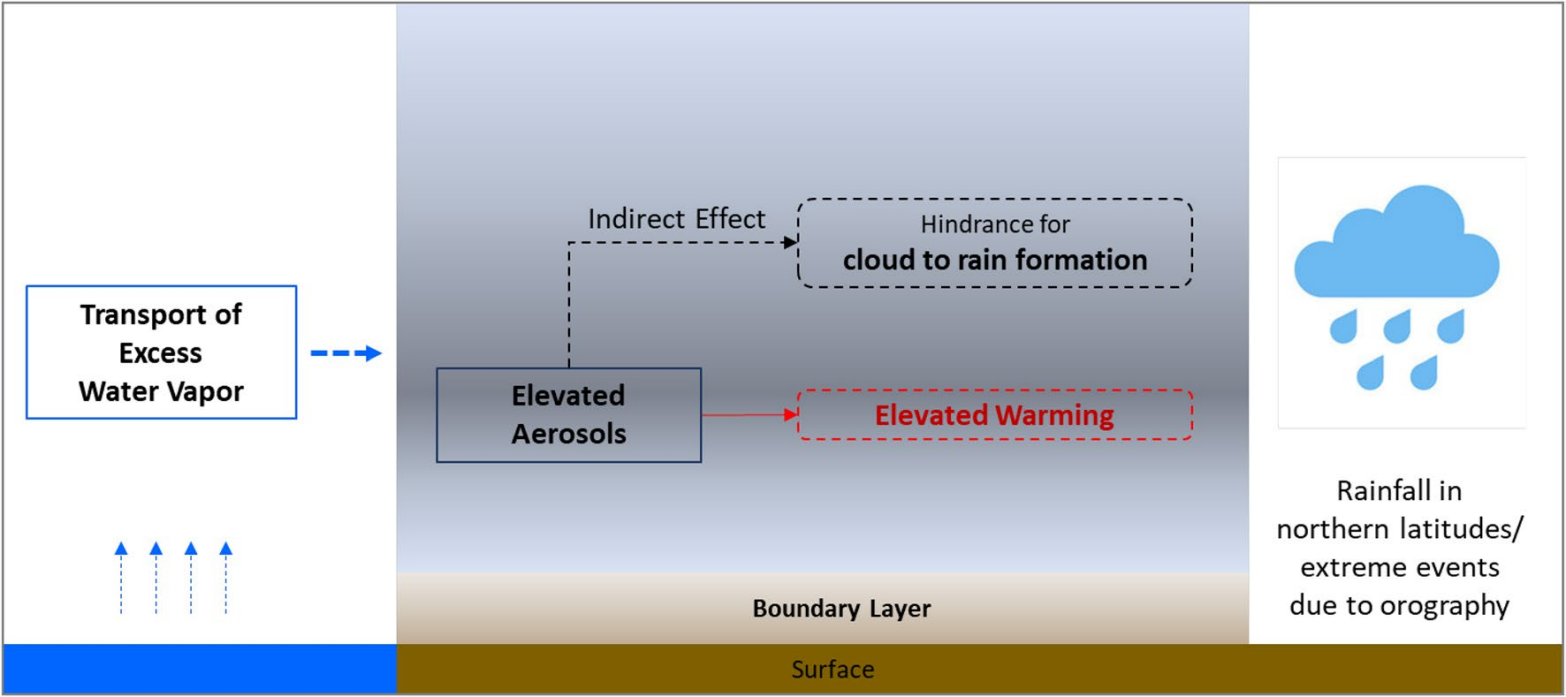
A pictorial representation of the proposed mechanism to be investigated. (The Figure is made using Microsoft Power Point).

The explained processes can be summarised in Fig. 10 which shows that the elevated layer of absorbing aerosols not only modifies cloud characteristics, but also reduce the convective strength impacting cloud formation and cloud-to-rain conversion. This process along with the background meteorology leads to a deficiency of rain in the usual high rain zones. The excess water vapor from these zones can be carried by background circulation and produces rain at northern orographic barriers like the Himalayan region^55^ where the water vapor can accumulate and produce convective or orographic rain.

However, our analysis is limited to data from 2000 onwards due to the availability of satellite (MODIS) aerosol data as against of requirement of analyzing data over a longer period to establish such trends and the possible causes. Future studies with more extensive observations fusing numerical models and satellite data could lead to more robust analyses. Additionally, while the MLR technique used here is a valuable tool, the availability of more data would increase the reliability of the model. In the present study, we provide a qualitative understanding of the probable factors influencing precipitation anomalies.

In the case of aerosols above the boundary layer that hinder convection and affect cloud formation, the indirect effects of aerosols also need to be investigated in detail to understand the aerosol impact on the precipitation change in recent years. This needs a dedicated modeling study focusing on highly impacted regions like the Bay of Bengal that could quantify the precise role of aerosols altering temperature profiles and cloud hydrometeors.

Additionally, this study shows only a hint of a probable change in circulation (e.g. the 2019 wind pattern at 850 hPa is different from the mean wind pattern of 2000–2019 in Fig. 6) which needs to be studied over the globe to understand the role of the circulation on the precipitation. Overall, this work aims to highlight the changing precipitation dynamics in South Asia. Unlike traditional views suggesting increased water vapor will result in the “dry gets drier, wet gets wetter” scenario, present findings reveal a more complex scenario where precipitation depends on a multitude of factors. The precise contribution of all the factors is complicated and can be solved through regional modeling which is the future goal of this research.

### Methodology

The study region is divided into 9 sub-regions namely, North West Region (NWR) near north-western India, Arabian Sea (AS), West Coast of India (WC), Bay of Bengal (BoB), Myanmar Coast (MC), Indo-Gangetic Plain (IGP), East Coast of India (EC), North-East India (NEI), and Tibetan plateau (TP) for easy reference (Fig. S1). Results are mostly described in terms of these sub-regions. The long-term precipitation anomaly trend along with mean precipitation has been studied for the Indian region (5^°^S-45^°^N;55^°^E-105^°^E) using long-term Global Precipitation Climatology Project (GPCP) Version 2.3 data^56^ for the pre-monsoon months (Fig. 1). The GPCP consists of monthly satellite-gauge and associated precipitation error estimates and covers the period January 1979 to the present. GPCP combine the precipitation information available from each of several satellite and in situ sources into a final merged product, using three types of measurements: passive Microwave estimates based on polar-orbit passive microwave satellites using Special Sensor Microwave Imager (SSMI) data; infrared precipitation estimates using Geostationary Operational Environmental Satellite (GOES) data and Polar Orbiting Environmental Satellites (POES) data; as well as other low earth orbit data and in-situ observations. Data are provided on a 2.5-degree grid^57^. The rainfall anomaly trend is calculated over a long period of 20 years (2000–2019) and the anomaly has been calculated concerning the mean rainfall data of 30 years from 1981 to 2010. It may be noted that, as the aerosol data (MODIS AOD) is only available from 2000, most of the parameters are considered for that period (2000–2019). For precipitation, considerably larger dataset from GPCP is used and the period spanning from 1981 to 2010 serves as the baseline for calculating pre-monsoon precipitation anomalies during 2000–2019.

Linear regression has been employed to find the trend and significance has been tested using the Mann–Kendall method. The daily rainfall is also taken from the GPCP 1DD product from the ERA-5 data archive with a grid size of 1^° ^× 1^°^^58^. In Fig. 2 high rainfall days are defined as rainfall within a day > 6 mm.dy^−1^ and low rainfall days are defined as daily rainfall < 2 mm.dy^−1^. The number of days with both criteria is calculated as the percentage of days with rainfall > 0 mm.dy^−1^.

Daily OLR data has been taken from the NOAA data archive with a grid resolution of (2.5^° ^× 2.5°). Outgoing Longwave Radiation (OLR) data at the top of the atmosphere are observed from the AVHRR instrument aboard the NOAA polar-orbiting spacecraft^59^. NOAA Daily Outgoing Longwave Radiation (OLR) data provided by the NOAA PSL, Boulder, Colorado, USA, from their website at https://psl.noaa.gov. The OLR anomaly was calculated with reference to the average value calculated from 1981 to 2010. The linear trend is calculated within the same period (2000–2019) as the precipitation.

Aerosol optical depth (AOD) at 550 nm data taken from the Moderate Resolution Imaging Spectroradiometer (MODIS) level 3 collection 6.1 monthly data set for resolution of 1^° ^× 1^°^. The deep blue (DB) and dark target (DT) combined products have been used for reliability^60^. The DT algorithm is used over land and ocean, and DB algorithms over land. The DT algorithm was developed to retrieve aerosols over dark-target surfaces, while DB algorithms were mainly designed to overcome the flaws in the DT algorithm over bright surfaces. MODIS C6.1 aerosol has several improvements to the previous C6 products^61^. The C6.1 DB algorithm has detected heavy smoke, reduced artefacts in heterogeneous terrains, improved surface modeling in elevated terrains, and updated regional or seasonal aerosol models over land. Moreover, to improve the data coverage, a new combined DT and DB (DTB) dataset is introduced over land according to independent MODIS monthly NDVI products by a simple approach that leverages the strengths of DT and DB algorithms^62^.

The Cloud-Aerosol Lidar and Infrared Pathfinder Satellite Observations (CALIPSO) is a satellite developed by NASA and CNES for the monitoring of cloud and aerosol properties^62^. It carries the Cloud-Aerosol Lidar with Orthogonal Polarization (CALIOP), an active polarization-sensitive lidar instrument that produces vertically resolved images of cloud and aerosol layers as well as their respective optical properties^62^. CALIPSO can reliably detect aerosol over bright surfaces, e.g. deserts, and beneath thin clouds. Measurements are taken both in the daytime and in the night-time parts of the track. The data is available online from NASA (https://asdc.larc.nasa.gov/project/CALIPSO/CAL_LID_L3_Tropospheric_APro_CloudFree-Standard-V4-20_V4-20). In this study, the aerosol profile information has been gathered from CALIPSO level 3 data with 2.5^° ^× 2.5^°^ resolution. CALIPSO differentiates the data into 3 major types of aerosols, dust, polluted dust, and smoke. These three types along with the extinction profile for all types of aerosol at 550 nm are integrated over a height range of 1.6–4 km. the integrated values for each month are used for the monthly average and trend information. CALIPSO and MODIS data have been used for the time period of 2000–2019.

Water vapour and cloud fraction data acquired from the MODIS level 3 data. Water vapour measured by the MODIS in near infrared within the cloud pixel (Water_Vapor_Near_Infrared_Cloud_Mean_Mean) and cloud fraction product of MODIS is “Cloud_Fraction_Mean_Mean”. Cloud hydrometeor data from single layer cloud is taken from the product, Cloud_Water_Path_1L_Liquid_Mean_Mean, and Cloud_Water_Path_1L_Ice_Mean_Mean. To find the ratio of water vapor to cloud hydrometeor amount (IWC + CWC in gm.m^−2^) the water vapor data (cm) is converted into gm.m^−2^(1 cm = 10^4^ gm.m^−2^) and then the ratio is calculated.

ERA-5 pressure level data has been used to calculate the RH and Temperature mean and trend for the near surface measurements (1000 mb). The streamlines have been plotted using horizontal and vertical wind speed information at 850 mb. Additionally, vertical velocity (Pa.s^−1^) from ERA-5 has been converted into vertical wind velocity (m.s^−1^) for the analysis.

To identify any parameters controlling the rain anomaly, multivariate linear regression (MLR) analysis has been performed over regions with significant rain variability as seen in the above study. The wind (W), relative humidity (RH), temperature (T) and AOD are taken as independent variables, and precipitation (P) is taken as a dependent variable. The basic equation for the linear model is given below.

$$P= {\beta }_{0 }+{\beta }_{1 }.W+{\beta }_{2 }. RH+ {\beta }_{3 }.T+ {\beta }_{4 }.AOD+\varepsilon$$

where magnitude of ${\beta }_{n}$ indicates the impact of the controlling parameters whereas the sign indicates the nature of correlation between precipitation and the parameters during the study period of 2000–2019. The model validity was tested based on the determination coefficient (R^2^), and *p*-value of the fitting. The best-fitted linear models are plotted in the Fig. S8 where it can be seen that the combination of all controlling factors provides a quite reliable linear model to indicate the precipitation scenario over the Indian region.

### Supplementary Information

Supplementary Figures.

## Data Availability

All the datasets used in this study are open-source data: Global Precipitation Climatology Project (GPCP) Version 2.3 (https://psl.noaa.gov/data/gridded/data.gpcp.html) and GPCP 1DD product from the ERA-5 data archive (https://cds.climate.copernicus.eu/cdsapp#!/dataset/satellite-precipitation?tab=overview). Daily OLR data has been taken from the NOAA data archive (https://psl.noaa.gov). Aerosol optical depth (AOD) at 550 nm data taken from the Moderate Resolution Imaging Spectroradiometer (MODIS) level 3 collection 6.1 monthly data set (https://ladsweb.modaps.eosdis.nasa.gov/). CALIPSO datasets are obtained from: https://asdc.larc.nasa.gov/project/CALIPSO/CAL_LID_L3_Tropospheric_APro_CloudFree-Standard-V4-20_V4-20). Background meteorological data are taken from the ERA-5 pressure level data (https://cds.climate.copernicus.eu/cdsapp#!/dataset/reanalysis-era5-pressure-levels-monthly-means?tab=overview). SST data is taken from https://www.psl.noaa.gov/data/gridded/data.noaa.oisst.v2.highres.html. Salinity and ocean heat content data taken from: https://cds.climate.copernicus.eu/cdsapp#!/dataset/reanalysis-oras5?tab=overview.
