# Supplementary material for: Change in precipitation pattern over South Asia in response to the trends in regional warming and free-tropospheric aerosol loading
Source: Sci Rep. 2024 Jun 24;14:14528. doi: 10.1038/s41598-024-64842-7 (PMC11196666; doi:10.1038/s41598-024-64842-7)
Supplement: Supplementary file 1 — Supplementary Figures. [file 41598_2024_64842_MOESM1_ESM.docx]

**Supplementary Figures:**


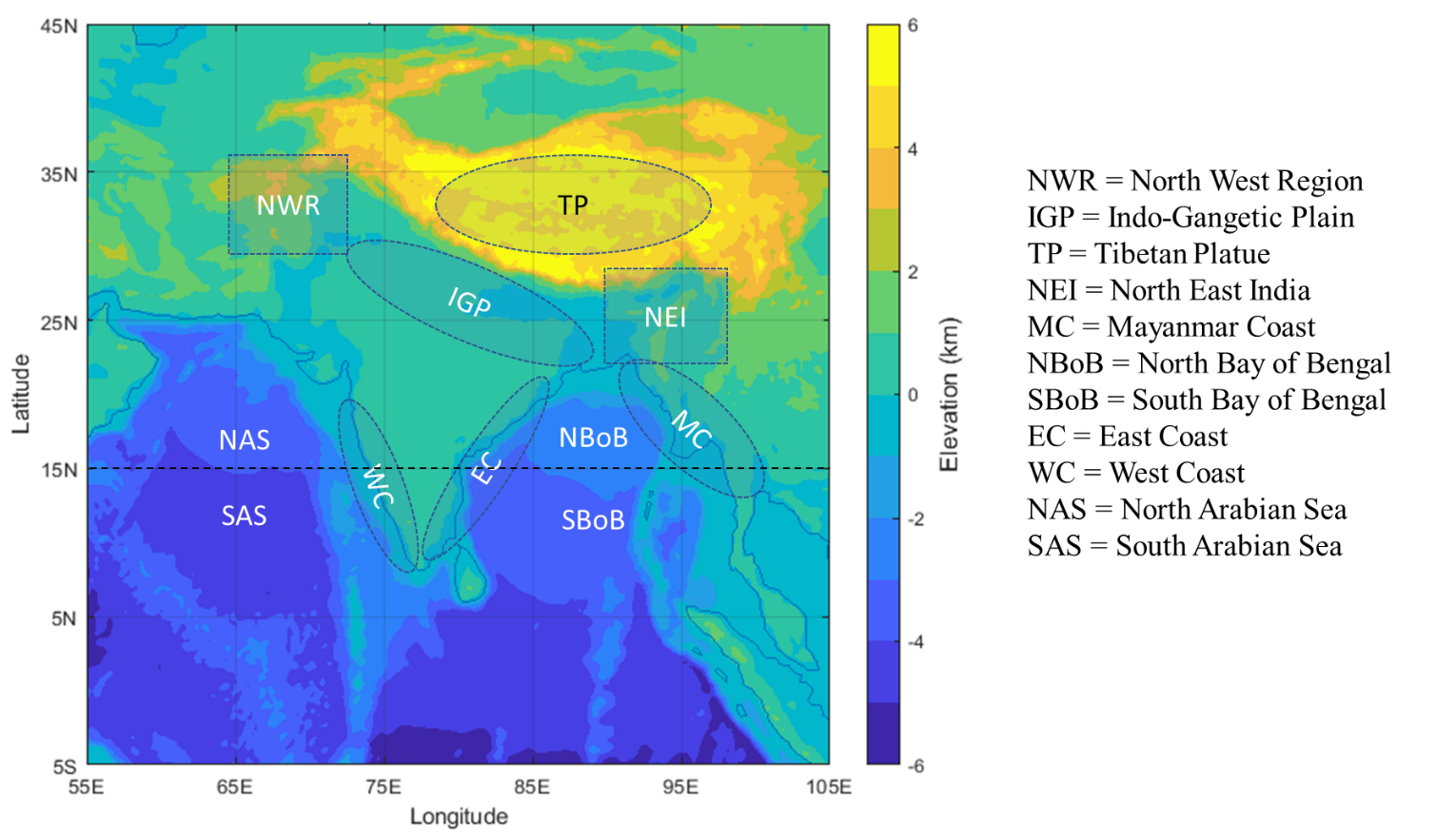


Figure S1: The subdivisions of the study region for ease of reference. The elevation data has been used from <http://research.jisao.washington.edu/data_sets/elevation/>. The Figure is made using MATLAB version 9.13.0 (R2022b), The MathWorks Inc. <https://www.mathworks.com>.

Figure S2. Average (2000-2019) cloud fraction during (a) March, (b) April, and (c) May; and trend in cloud fraction during (d) March, (e) April, and (f) May. The dots correspond to a > 90% significance level using the Mann-Kendall method. The Figure is made using MATLAB version 9.13.0 (R2022b), The MathWorks Inc. <https://www.mathworks.com>.

Figure S3. Average Dust AOD (1.6-4 km) in (a) March, (b) April, and (c) May during 2000-2019 and trend in dust AOD in (d) March, (e) April, and (f) May for the same period. The Figure is made using MATLAB version 9.13.0 (R2022b), The MathWorks Inc. <https://www.mathworks.com>.

**Trend in Polluted Dust AOD, Mar**

(d)

(b)

(e)

**Trend in Polluted Dust AOD, Apr**

(a)

(f)

**Trend in Polluted Dust AOD, May**

Latitude (^o^)

(c)

Longitude (^o^)

Figure S4. Average (2000-2019) Polluted Dust AOD (1.6-4 km) during (a) March, (b) April, and (c) May and trend in polluted dust AOD in (d) March, (e) April, and (f) May. The Figure is made using MATLAB version 9.13.0 (R2022b), The MathWorks Inc. <https://www.mathworks.com>.

Figure S5. Average (2000-2019) Smoke AOD (1.6-4 km) during (a) March, (b) April, and (c) May and trend in smoke AOD in (d) March, (e) April, and (f) May. The Figure is made using MATLAB version 9.13.0 (R2022b), The MathWorks Inc. <https://www.mathworks.com>.


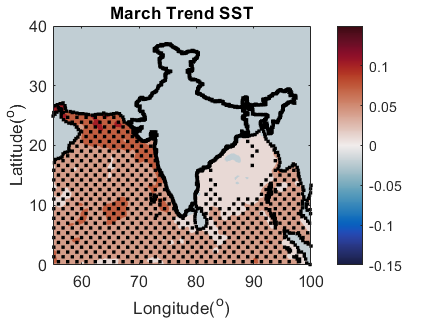

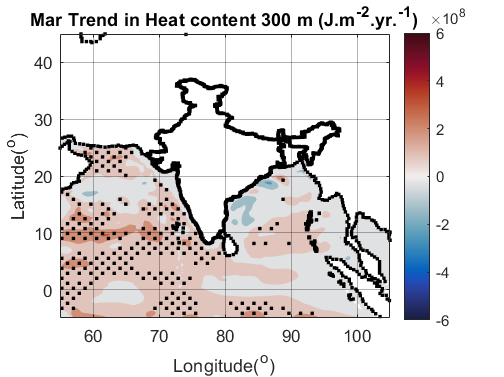

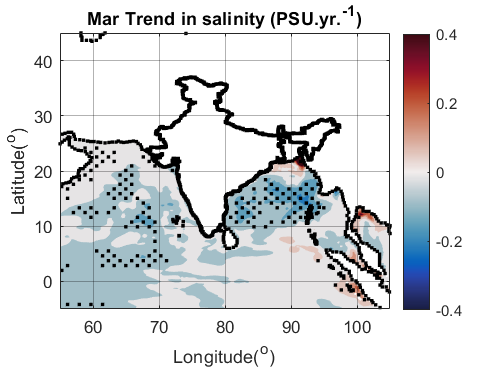

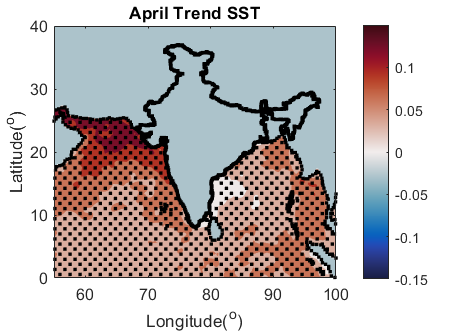

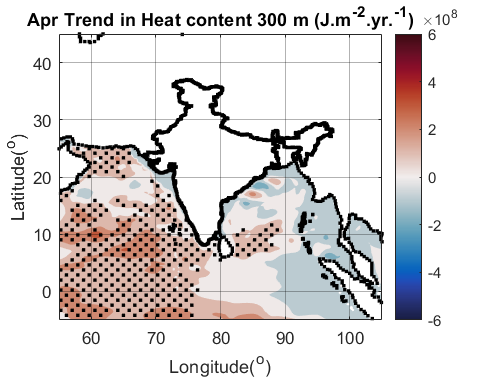

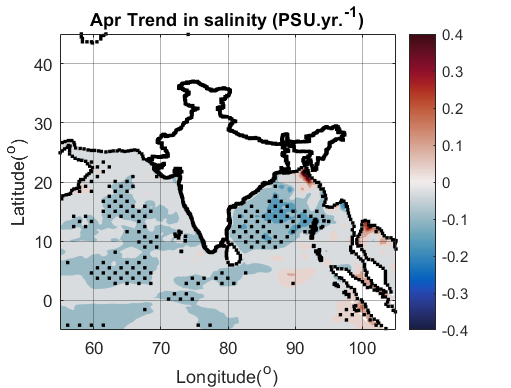


(g)

(a)

(d)

(b)

(c)

(e)

(i)

(f)

(h)

(d)


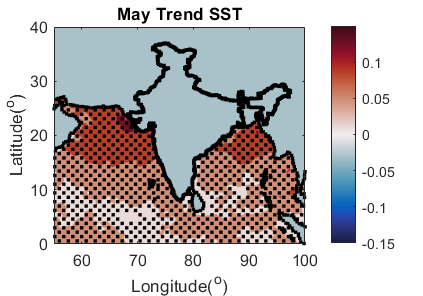

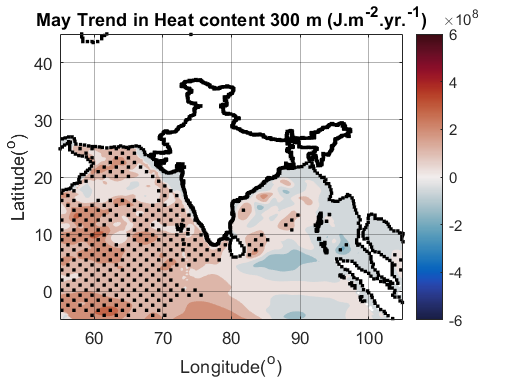

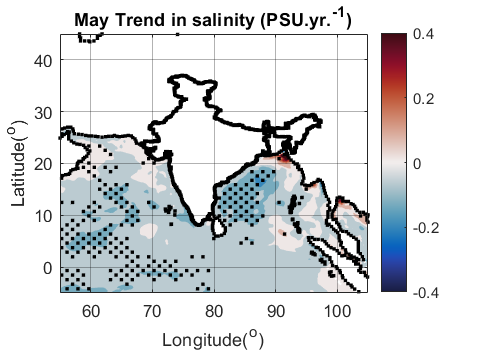


Figure S6. Anomaly trend in SST (K.yr^-1^), OHC, and salinity (PSU) during 2000-2019 for March, April, and May. The Figure is made using MATLAB version 9.13.0 (R2022b), The MathWorks Inc. <https://www.mathworks.com>.

**Reg. 1**

**Reg. 3**

**Reg. 2**

**Reg. 4**

Figure S7: The selected region for multilinear regression analysis: (a) Region 1 (90°-100°E; 6°-22°N), (b) Region 2 (64°-74°E; 25°-34°N), (c) Region 3 (63°-77°E; 8°-20°N), and (d) Region 4 (88°-98°E, 23°-31°N). The Figure is made using MATLAB version 9.13.0 (R2022b), The MathWorks Inc. <https://www.mathworks.com>.

(a)

(b)

Figure S8: Linear models (a) with AOD taking into accountand (b) without AOD. Their performance is based on R^2^ and p-value for different regions for pre-monsoon months. The Figure is made using MATLAB version 9.13.0 (R2022b), The MathWorks Inc. <https://www.mathworks.com>.
